# Governing biotechnology to provide safety and security and address ethical, legal, and social implications

**DOI:** 10.3389/fgene.2022.1052371

**Published:** 2023-01-11

**Authors:** Benjamin Trump, Christopher Cummings, Kasia Klasa, Stephanie Galaitsi, Igor Linkov

**Affiliations:** ^1^ Engineering Research and Development Center, United States Army Corps of Engineers, Vicksburg, MS, United States; ^2^ Genetic Engineering and Society, North Carolina State University, Raleigh, NC, United States; ^3^ Gene Edited Foods Project, Iowa State University, Ames, IA, United States; ^4^ Department of Healthcare Management and Policy, University of Michigan School of Public Health, Ann Arbor, MI, United States; ^5^ Department of Engineering and Public Policy, Carnegie Mellon University, Pittsburgh, PA, United States

**Keywords:** biotechnology, ELSI, policy, governance, safety-by-design, synthetic biology

## Abstract

The field of biotechnology has produced a wide variety of materials and products which are rapidly entering the commercial marketplace. While many developments promise revolutionary benefits, some of them pose uncertain or largely untested risks and may spur debate, consternation, and outrage from individuals and groups who may be affected by their development and use. In this paper we show that the success of any advanced genetic development and usage requires that the creators establish technical soundness, ensure safety and security, and transparently represent the product’s ethical, legal, and social implications (ELSI). We further identify how failures to address ELSI can manifest as significant roadblocks to product acceptance and adoption and advocate for use of the “safety-by-design” governance philosophy. This approach requires addressing risk *and* ELSI needs early and often in the technology development process to support innovation while providing security and safety for workers, the public, and the broader environment. This paper identifies and evaluates major ELSI challenges and perspectives to suggest a methodology for implementing safety-by-design in a manner consistent with local institutions and politics. We anticipate the need for safety-by-design approach to grow and permeate biotechnology governance structures as the field expands in scientific and technological complexity, increases in public attention and prominence, and further impacts human health and the environment.

## 1 Introduction

Science and technology innovation are both constant and essential for companies and countries to survive and compete. Nanotechnology, synthetic biology, and artificial intelligence are a small subset of innovation that may disrupt and transform all facets of life, ranging from communication and the workplace to medicine and industry. With timelines ranging from five to 50 years, countries and companies are competing to be the first actors capable of transforming emerging science into in-demand commodities and mechanisms to achieve national objectives. This goal, described colloquially as “technology modernization” or “technology advancement,” is a construct that encapsulates a process whereby existing science and technology capabilities are replaced or complemented by new practices, materials, or equipment that infer a range of possible benefits to the end-user. For instance, in September 2022, President Biden signed an Executive Order to mandate that the United States invest significant resources to “harness the full potential of biotechnology and biomanufacturing” which will expand market opportunities, train a more diverse skilled workforce, streamline regulations, and advance biosafety and biosecurity initiatives (White House, 2022). In this vein, the majority of investment seeks to expand the field by identifying the physical, chemical, or biological properties and operations which would theoretically enable breakthroughs. However, as in cases such as the United States *via* Executive Order 14,801, there is present need to identify, evaluate, and mitigate potential limitations that may hinder safe and successful biotechnology modernization efforts (White House 2022).

While debates related to the end-goals of technology development abound, far less research and scholarship is dedicated to the developmental challenges and rate-limiting steps to successfully advance and harness such cutting-edge science for useful purposes. A small but rising level of discourse has centered upon the social sciences, including studies in risk, governance, and ELSI (ethical, legal, and social implications) (Greenbaum 2015). Few mechanisms glue the physical and social science explorations together into a single narrative of technology development, and this contributes to a lack of cohesion regarding emerging technology governance (Trump et al., 2019). Such limitations generate considerable resistance to technology modernization (Pauwels 2013; Florin and Bürkler, 2017). While scientific advancement and technological innovation at the bench yields new products, infrastructure, and capabilities to improve various facets of life, a critical question remains for the United States and other countries—will companies and the public trust, use, and appreciate such innovation? Alternatively, will their actions prevent scientific developments and their associated products from becoming politically, economically, and socially sustainable?

The implications of these questions are hotly contested in the field of biotechnology (Douglas and Stemerding, 2014; Mandel and Marchant, 2014). How do we know when a product is safe? What are the potential indirect or unintended consequences to various receptors? What safeguards can help mitigate hazards? What mechanisms or institutions can help monitor, test, and remediate harmful consequences? These are but a small selection of concerns raised during the early debates around genetically modified organisms (GMOs) that are being amplified within modern biotechnology research and strategic goals.

Some longstanding areas of research, such as genetically modified agriculture, have been subject to numerous restrictions in the European Union, Australia, and beyond based upon social concern as much as risk-based inquiry (Levidow et al., 2000; Guehlstor and Hallstrom, 2005; Murphy et al., 2006). For example, genetically modified (GM) plants, specifically crops, were originally developed to improve crop protection, and produce other benefits such as higher yields, greater durability, and increased nutrition. But, a lack of public engagement from large biotechnology corporations and governmental bodies, poor public understanding of what biotechnology and genetic engineering are, and public fears of the short and long-term risks of GM plants on human and environmental health led to massive backlash against them throughout the 1990s and early 2000s (Williams 1998; Butler et al., 1999; Varzakas et al., 2007; Gaskell et al., 2010; Blancke et al., 2015). Biotechnology companies have slowly started to learn from their mistakes, realizing that consumer activism is important on product success or failure, as well as understanding that a focus should be placed on highlighting the positive aspects of a technology instead of only reacting to negative backlash (Mohorčich and Reese, 2019). Additionally, public trust is critical for technology success. Unfortunately, existing restrictions against GM development limit the benefits that these biotechnologies can provide in a world where starvation is still a common problem. Other areas such as human health research similarly lack consistent strategies to prioritize breakthrough applications that can prevent, mitigate, or cure humanity’s various ills (Kuzma and Tanji, 2010; Greer and Trump, 2019). A system is needed to ensure that the risks presented by such technological innovations are evaluated early and often, such that the products that are ultimately developed are palatable for the societies that will be using them.

While virtually all emerging technologies encounter various risk, governance, and ELSI-centered hurdles and roadblocks along their developmental path, biotechnology is perhaps most broadly affected by such limitations. Extensive regulatory review times (e.g., the AquaAdvantage Salmon) as well as existential political and/or social reluctance to approve and commodify biotechnology products leaves the field in many countries on an unsustainable trajectory reliant upon large government grants as opposed to bottom-up industry funding and public profitability. This serves to limit the technology’s expansion and potential benefits from reaching society. Consequentially, countries are unable to innovate and grow because they i) continue to lack the mechanisms to establish safe use requirements and practices for such products, and ii) have limited understanding of the security-based challenges associated with potential technological misuse in a domestic or foreign environment, resulting in higher vulnerability to misuse across commercially developed biotechnologies.

While biotechnology advancements are lauded for their potential benefits to society, great care must be taken to ensure that modernization efforts prioritize safety and security, as well as the ELSI for how they are produced, disseminated, used, disposed of, and governed (Jansen and Gupta, 2009; Titus et al., 2020). For example, in 2018, a biotechnology experiment resulted in the birth of twin girls reported immune to HIV following manipulations to the embryos using CRISPR—clustered regularly interspaced short palindromic repeats used as a technology to edit genes. This isolated case, its process and its results open up a “Pandora’s Box” of concerns and dilemmas regarding misuses of biotechnology (Raposo 2019). Potential applications of CRISPR and other genomic editing tools demonstrate vital needs for the development and coordination of biotechnology frameworks that prioritize and synthesize non-technical needs within traditional research and development pipelines. This paper will articulate the core needs for responsible biotechnology development which can be achieved through robust upstream prioritization of safety-by-design approaches that incorporate ELSI considerations and recent best practices following the TAPIC principles (transparency, accountability, participation, integrity, and capacity—Greer et al., 2020) of robust technology governance.

## 2 What does biotechnology modernization mean? Early thoughts on a growing problem

Technology modernization, also referred to as technology advancement, is a geopolitical construct as much as a scientific objective. On one hand, countries aspire towards technological modernization to secure the economic, health, defense, and homeland security benefits that stem from “first actor” and “early actor” privilege in science and technology leadership (Springut et al., 2011; Komkov et al., 2021). On the other, technology modernization is a natural extension of scientific capabilities—our understanding of science can always expand and improve, and make its resulting technologies faster, cheaper, or more generally superior to current offerings. The former is a more normative and strategic mission, while the latter is borne from human curiosity and ingenuity. This piece focuses upon the more normative and aspirational intent behind geopolitically driven biotechnology modernization, where many countries are currently competing for scientific advancement and dominance in the coming years and decades.

In present-day geopolitical framing, biotechnology modernization is an attempt to expand and harness the immense opportunities generated from scientific breakthroughs in the 1990s through the 2010s. From the success of the Human Genome Project (Watson, 1990) to more recent breakthroughs related to clustered regularly interspaced short palindromic repeats (CRISPR) within a genome editing process, potential benefits from successful technology development range from evolutionary (incremental improvements over existing conventional products or materials) to revolutionary (significant advancement in material properties and/or delivery of capabilities not currently accessible to the public). Governments seek to gain leadership over both end-goals within their modernization plans yet are particularly interested in providing resources and institutional capabilities to foster disruptive and strategic advantage.

Disruptive technological change can occur in a variety of applications. For much of human history, technological advancement was fundamentally a military or economic concern, with both objectives often intertwined. Biotechnology, however, diverges from other technological development campaigns by the late-20th Century, where offensive applications of biological research (e.g., “biological weapons” research *via* engineered or adapted viruses or bacteria) became socially unacceptable and geopolitically deprioritized *via* the Biological Weapons Convention (signed 1972, in force 1975) (Millett, 2010). While multiple countries (e.g., apartheid South Africa, Hussein-era Iraq, the Soviet Union, etc.) and non-governmental organizations (e.g., Aum Shinrikyo) possessed or sought offensive biotechnological capabilities, greater emphasis was placed upon commercial research and biological safety initiatives, such as with the Asilomar Conference on Recombinant DNA (1975) or the Cartagena Protocol on Biosafety (in force 2003) (Krimsky, 2005). Discussion by this point emphasized the need to foster norms and develop best practices, risk-informed safety procedures and overall guidance regarding human safety and biodiversity protection in the pursuit of genetic engineering and biotechnology advancement.

By 2020, such principles are core considerations of ongoing biotechnology modernization discussion, but are no longer the driving force behind biotechnology’s scientific, institutional, or political discussion. In the early 2000s, a paradigm shift was triggered by advances in biological sciences and systems engineering, driven by an increasing understanding of the structure and operations across the genome alongside growing trends towards faster, cheaper, and de-skilled approaches for genetic engineering (i.e., “synthetic biology”) (Cameron et al., 2014). Internationally, governments have increasingly grown interested in harnessing the potential of biotechnology in all applications including defense investments in a range of non-weapon applications. Initial successes included the construction of a *Mycoplasma mycoides* genome that was subsequently transplanted into a *M. capricolum* recipient cell whose genome had been removed, demonstrating early capacity to “build” rather than “evolve” life (Gibson et al., 2010). Other advancements included the demonstration of CRISPR as a genetic engineering technique involving a *Cas9* nuclease that, along with a synthetic guide RNA (gRNA), can cut a cell’s genome at a desired location to foster the more precise addition, deletion, insertion, or substitution of genes or suites of genes (Doudna and Charpentier, 2014; Chen et al., 2017). Such sweeping advancements in modern biology and genetics shifted discussion from applying principles of precaution to genetic engineering research of the 1980s and 1990s to a more fast-paced yet tenuous race to unlock future sweeping advances towards novel scientific capabilities.

While large and well-resourced laboratories dominate the field’s cutting edge, a groundswell of activity from dozens of countries and hundreds of universities, secondary schools, and individual DIY “biohackers” have demonstrated two core realities for biotechnology modernization: first, biotechnology will inevitably diversify in a manner similar to computer programming and information systems development *via* Silicon Valley, and should be expected to yield new opportunities and products onto the world. Second, and perhaps far more consequential, is that it is no longer possible to restrict access to much of biotechnology’s emerging capabilities to a relatively small and well-governed subset of graduate-trained and institutionally funded scientists, introducing a broad host of safety and security questions about the current path of the field in the future. The top-down governance and strategic development of biotechnology in decades past will need to contend with the less structured and more unpredictable bottom-up interests from countries, companies, groups, and individuals that historically have had little access to biotechnology research capabilities. There is a wealth of decentralized, market-driven biotechnological development occurring that is less amenable to using traditional, often government-driven, top-down efforts to raise ELSI issues.

As such, the future of biotechnology modernization characterized by constant competition and accelerating technical capacities in research. Strengthening trends indicate that emerging biotechnologies are increasingly affordable (machinery and products), having fewer barriers to entry, are intellectually accessible, and sufficiently globalized in a manner where any interested actor could meaningfully participate. In turn, the future success of biotechnology modernization will be determined by the willingness of governments, companies, and organizations to.

a) foster a scientifically-literate workforce
b) acquire and maintain critical infrastructure, machinery, and resources
c) develop and achieve core objectives regarding scientific mastery and technological deployment and
d) craft a suitable governance culture that sustains and advances future biotechnology aims and capabilities.

At the core these actions are the ability to identify needs and opportunities and quickly adapt organizational capabilities to meet emerging objectives. This is often far easier for smaller and more focused companies and non-governmental organizations than for large government research entities that are responsive to additional layers of political and institutional risk culture (Douglas and Wildavsky 1983).

For the period from 2022–2032, biotechnology modernization will be defined by the inclusion of sweeping technological advancement where inventors and developers will have significant “first actor” privileges. However, incentives to unlock longstanding unattained objectives–curing or preventing debilitating disease, fostering sustainable energy, preserving biodiversity and improving environmental conditions, among others—will generate intense competition. No country or entity will fully dominate the technological landscape, yet those better able to adapt and seize new opportunities will have the greatest opportunity of sustaining leadership in their field. For governments looking to facilitate biotechnology modernization, adaptivity is predicated upon two needs: i) the ability to identify opportunities in the science and technology space, and ii) the capacity to demonstrate safe, secure, ethical, and equitable characteristics that helps address a variety of social roadblocks to further technology development, ranging from regulatory approval requirements to public trust and participation within the future bioeconomy. Both concepts are further discussed in sections below.

## 3 Rough projections of the future: Aspirations for future biotechnology

Though biotechnology modernization is a socially complex idea, it is rooted in a single objective: ensuring that future technological offerings are superior to those currently available. Scientifically, this objective requires a cursory understanding of the technological abilities that might be achieved in the coming decades, given various levels of effort and resourcing. Among the more famous examples of this include the Manhattan Project (1942–1946), the Apollo program (1961–1972), and the Human Genome Project (1990–2003). Regardless of the motivations behind these and other efforts, modernization effort should move efficaciously towards a set goal, whose progress will give the first actor a range of desirable benefits.

Biotechnology modernization is as much a series of scientific and technical achievements as it is a series of normative advancements. While social sciences literature framed the benefits and disruptive implications of emerging biotechnologies such as engineered biofuels or novel therapeutics in previous decades, scientists, engineers, and programmers have pursued more technical objectives that could render transparent, safe, and repeatable practices at the bench. Critical advancements have ranged from an improved understanding and refined degree of control over the manipulation of an organism’s genome, to the application of new techniques to foster improved medical and industrial products.

While various permutations of emerging biotechnologies exist, a synopsis of their applications are described in Table 1 below (Wang et al., 2010; Clark and Pazdernik, 2015).

**TABLE 1:** Key biotechnology industries and applications.

Biotechnology industry	Description of application
Industrial Biotechnology	Application of biotechnology for streamlined production and refinement of industrial materials (e.g., the development of enzymes as catalysts to produce, modify, or remediate specific chemical substances)
Environmental Biotechnology	Application of biotechnology capabilities to remediate contaminants in the environment, facilitate biodiversity and protection of at-risk speciesetc.
Medical Biotechnology	Application of biotechnology to foster new therapeutics, medical procedures, or medical devices for the advancement of human health
Agricultural Biotechnology	Application of biotechnology to amplify available crop production processes (e.g., engineering transgenic plants suitable for specific environments or consumption targets)
Marine Biotechnology	Application of biotechnology to engineer, improve, or utilize sea resources in a sustainable fashion (e.g., engineered algae for algal ethanol and oil byproducts)
Informatics	Synthesis of computational and biological sciences to better understand biological data, particularly in the field of genomics, to better understand the organizational principles of the genome

Each of these fields possesses various endpoints that, by 2030, could present substantial technological advancement in medicine, industry, and national defense. Yet, these aspirations do not exist in a vacuum. In the real world, actors are frequently obligated to balance future benefits against a range of possible threats to humans, the environment, and broader safety and security (Oye, 2012; Redford et al., 2013). Much of biotechnology is fundamentally “dual-use” in that its development can equally enable normatively beneficial and nefarious purposes, all depending upon an actor’s perspective and motivations (Miller and Selgelid 2007; Marris et al., 2014; Cummings et al., 2021). For example, seemingly benign biological agents and viruses could be weaponized using genetic engineering technologies like CRISPR-Cas9 *via* gene drives (Himmel, 2019). Synthetic biology, digital biological data, and 3D printing could also be used for maleficent reasons. Likewise, even well-intended research may yield unforeseen hazards that may be particularly debilitating and even irreversible. As such, stakeholders in the biotechnology development process are forced to grapple with the ongoing challenge of which safeguards to implement in their research process to prevent malfeasance (“biosecurity”) and mitigate unintended hazards (biosafety) while not endangering the future success of a research effort (modernization) (Gronvall, 2019).

Different stakeholders hold diverging needs and motivations regarding biosafety and biosecurity. For one, profit-seeking companies are fundamentally driven to scientific innovation to unlock desired technologies and products whose potential payoff outweighs anticipated investment and risk. Such goals incentivize limiting safety-based concerns (e.g., regulatory disapproval of product, high insurance premiums, discouraging customer support) as well as biosecurity challenges (e.g., inability to trade with foreign partners, use of proprietary material in furtherance of a terrorist attack or war crime, theft of intellectual property, etc.). Likewise, government research agencies are less motivated by profit and are often well-resourced relative to their commercial partners across a range of biotechnology research ventures and are required to uphold stringent safety and security measures while adhering to political pressures and institutional objectives that may shift on a semi-regular basis. In this, companies and smaller organizations are more resource-starved yet far nimbler than government research agencies.

Such differences are compounded when accounting for social, cultural, political, and procedural differences that manifest at an international level. Governments like Singapore or the People’s Republic of China have demonstrated more adaptive capacity to shift institutional priorities and safety requirements than Western nations, where the inherent structure of governance as well as the diverging priorities for technological modernization foster competing incentives for differing countries (Greer and Trump, 2019). For example, human subjects research in China is executed within differing timelines, institutional barriers, and risk evaluations than in the European Union or the United States (Li et al., 2019). Likewise, the heated and multi-decade disputes about the perceived safety and consumer-driven need regarding GMOs in engineered agriculture in the United States and the European Union have contributed to diverging incentives and modernization pathways for these and other countries (Twardowski and Malyska, 2015; Hundleby and Harwood, 2019). These debates continue in the current generation of gene-edited food products (cisgenic editing) and media reports demonstrate distinct public calls for changes to governance structures in both the United States and European Union (Dalhstrom et al., 2022).

For democracies with longstanding biotechnology capacity and objectives, the globalization of biotechnology research makes it difficult to maintain world-leading status in a given vein of research while sustaining public trust, maintaining compliance with safety requirements, identifying and preventing security threats, and fulfilling political and institutional needs, expectations, and precedent. For such regimes, biotechnology modernization hinges upon the ability to avoid such tangled debates by eliminating or mitigating potential hazards as early as possible in the development process (“safety-by-design”), as well as fostering adaptive capacity to identify and ameliorate potential biosecurity threats. Such goals are beginning to develop in the scholarly literature yet have rarely been applied as tools of governance to foster public support for the future bioeconomy. Below, we discuss fundamental ideas on how such a balance may be achieved in a manner consistent with organizational and institutional expectations.

## 4 Achieving biotechnology modernization: Safety, security, and ELSI

Biotechnology modernization success depends upon two factors—unlocking technological capability and understanding all its potential implications, both beneficial and detrimental. The risks inherent in any new biotechnology applications must be assessed through the biosecurity, biosafety, and societal lenses to preclude any opportunity for the technology to create problems greater than those it is meant to solve. Biotechnology modernization must account for institutional requirements and cultural preferences of any population affected by the products commodification and deployment. Failing to do so will create challenges and roadblocks to technology adoption and beneficial usage.

### 4.1 Safety and biosafety

For the purpose of this paper, safety refers to the prevention of harms to people, while biosafety is specific to preventing harms caused by accidental or naturally occurring events involving biotechnology applications. Unsafe biotechnology products may provide opportunities to irresponsible actors to alter or mishandle them and inadvertently yield health or environmental hazards. Appropriate oversight mechanisms must prevent such technology misuse. Safety and biosafety considerations must align with existing standards, laws, and regulations, both domestically and internationally. In the United States, current regulations for biotechnology applications largely focus on product safety, rather than process development safety (Carter et al., 2014; National Academies of Sciences, Engineering, and Medicine, 2017; Trump, 2017). However, other forms of safety (e.g., workplace safety, packing and shipping, supply chain, etc.) should also be prioritized and executed following standards as set forth by OSHA and other regulatory bodies.

### 4.2 Security and biosecurity

For the purpose of this paper, security is defined as the capacity to insulate the system from disruptions that would otherwise interrupt functionality. We define biosecurity as aiming to prevent harm from the deliberate actions of nefarious individuals or negligent misuse of biotechnology applications. In the example of genetically engineered microbes, biosecurity concerns remain that the technology could be appropriated by an individual actor with malicious intent. Given the dual-use nature of emerging biotechnologies, nefarious actors might be able to engineer and deploy organisms with deliberately harmful properties to human and environmental health using critical knowledge and research capacity, which constitute “information hazards” (Lewis, et al., 2019; Nieuwenweg et al., 2021).

To address biosecurity concerns, the United States National Research Council established the Committee on Advances in Technology and the Prevention of their Application to Next-Generation Bioterrorism and Biological Warfare Threats, which has recommended a common culture of awareness and a shared sense of responsibility among life scientists (National Research Council, 2006). This recommendation emphasized the importance of establishing mechanisms of oversight for biological capabilities that could cause physical and/or environmental damage or have dual use applications, such as, hosting homeostatic and defense systems or for constructing synthetic organisms with limited control against their environmental persistence and spread, and/or the potential for deliberate negative health risk. Biosecurity efforts can complement biosafety efforts insomuch as they can be evaluated simultaneously, though strategies for improving each may vary. Effective biosecurity governance requires stakeholders to consider *future* security requirements; biosecurity governance requires the ability to anticipate potential dual use applications of the enabling technologies behind new biotechnology products and tools (Kuzma et al., 2018; Winickoff and Pfotenhauer, 2018) and mitigate opportunities for adversaries to misuse biotechnology research. As security is increasingly an international effort, the developers can supplement internal assessments by engaging with each other and specific government liaisons or agencies to obtain and share biosecurity threat information as it becomes available.

### 4.3 Ethical, legal, and social (ELSI) implications

The broader public makes values-based assessments and decisions regarding new technologies, which influence the technologies’ acceptance and potential for deployment. Developers must consider how and why diverse public domains may oppose a new technology’s application, which could erode public support for the mission and/or exacerbate international relations (National Academies of Sciences, Engineering, and Medicine, 2016). The following considerations parse the values-based concerns that could drive public opposition to a technology.

Ethics: The technology should prioritize the four principles of bioethics: 1) autonomy: recognizing individual rights and the importance of free will, 2) non-maleficence: never intentionally cause harm, including harm from negligence 3) beneficence: the duty to “do good.” and 4) justice: ensure fair distribution of benefits and costs across all individuals affected. The final principle, justice, has gained particular momentum recently as decision makers have increasingly recognized equity in planning and technology implementation. For example, one of the concerns of opponents of genetically modified crops is that its benefits largely accrue to producers while the risks fall mostly on consumers. Equitable biotechnology processes would strive to have risks and benefits borne by the same population.
Legal: Legal implications for biotechnology are rife with uncertainty. As with many new technologies, specific regulations for many biotechnology-enabled processes and products do not yet exist. For example, new regulations could stipulate a need to mitigate unintended spread of biotechnology products, appropriate barcoding of modified organisms, or the development of sensors and/or remediation agents in the event of unintended environmental release by a nefarious agent. However, the focus on *intention* in use has made many laws flexible, inclusive, and future-proofed in terms of regulating biological capabilities that are not specifically named. Often (but not universally), the intention of technology governance and law is crafted to “capture” many processes and products of a given technology and provide basic requirements for safe use and market entry. Developers must ensure compliance with regional laws and policies, and existing or evolving international accords as applicable. Its endurance shows the functional applicability of the law despite rapidly changing conditions.
Social: Critical views of biotechnology vary depending on cultural norms and societal taboos (e.g., cultural symbols, social values, dominant media frames). For instance, Pauwels (2013) found that values and trust significantly influence public perceptions of biotechnology. Critical views of biotechnology have also been observed among stakeholder groups where cultural beliefs influence views on biotechnology and gene editing (Kuzma and Cummings, 2021). Recent studies also find that social values, antecedent value dispositions, and media frames likely influence how the United States public views novel agrifood technologies like gene edited crops (Cummings and Peters, 2022; Dahlstrom et al., 2022). It is important to understand and to respect the various risk cultures (moral views and values within and across societies regarding the perceived risks and opportunities yielded by an emerging technology) where biotechnologies will be deployed.

**FIGURE 1:**
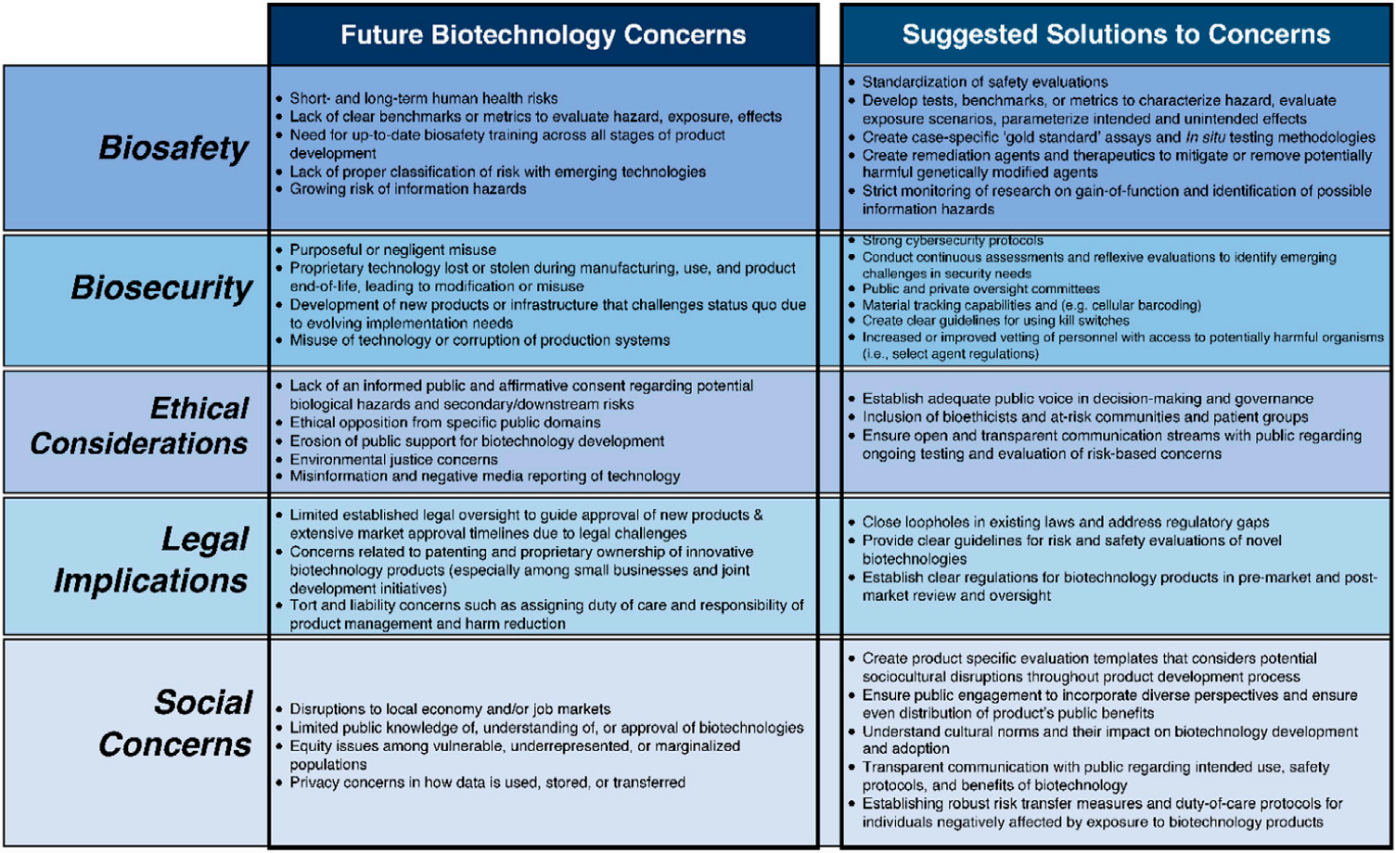
Examining ELSI concerns for expected future biotechnologies.

Collectively, these concerns characterize potential public backlash of any biotechnology deployment where the use of biotechnology may be opposed by certain groups and NGOs and may violate international laws and agreements (see Figure 1 for an in-depth overview of examples of ELSI concerns that may arise for future biotechnologies).

## 5 Governance frameworks for biotechnology

### 5.1 The TAPIC framework

**FIGURE 2:**
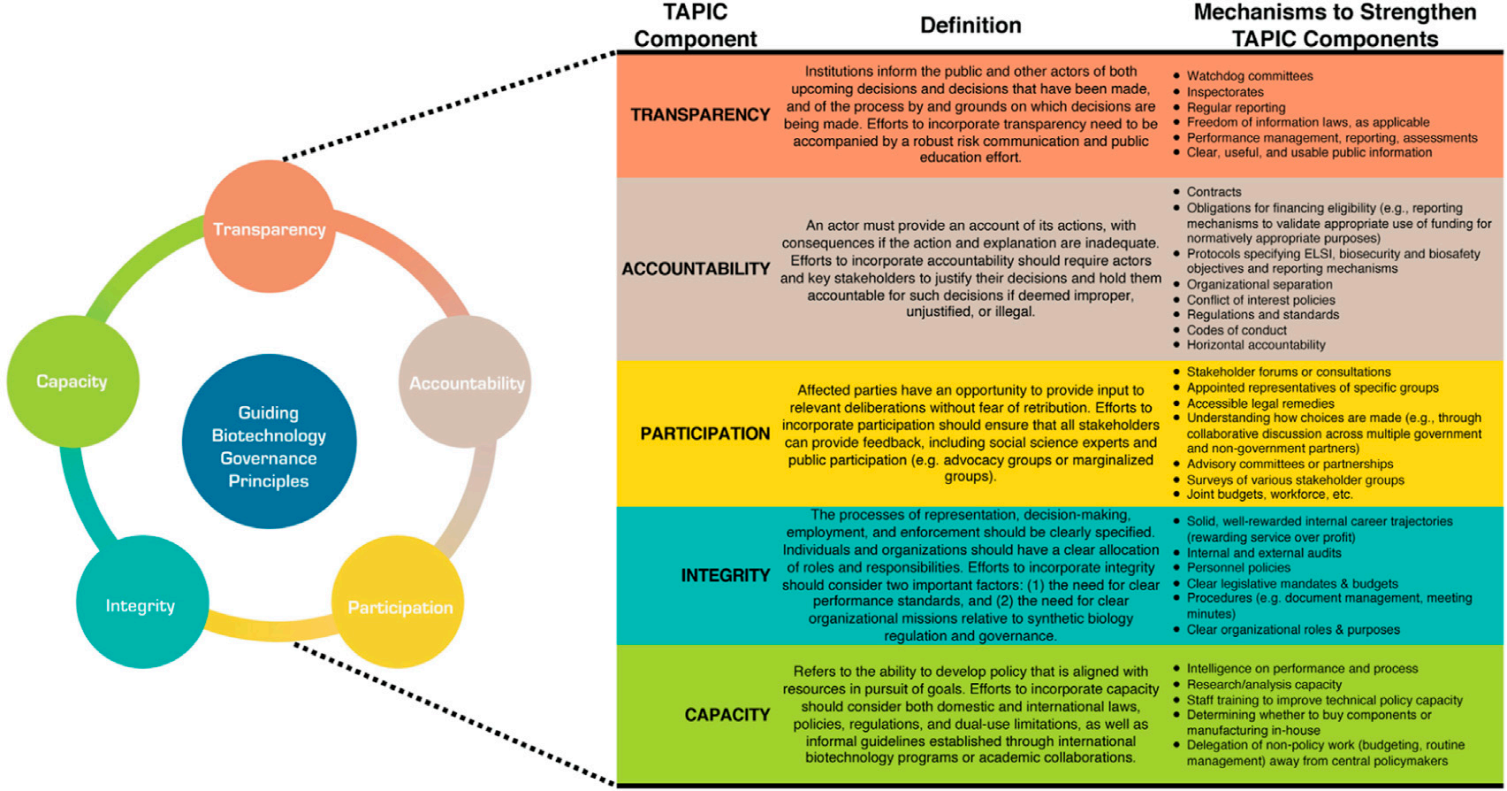
TAPIC framework for guiding biotechnology governance principles.

Addressing biosafety, biosecurity, and ELSI considerations related to biotechnology research and manufacturing requires collaboration and cooperation across multiple stakeholders spanning academic, government, industry, and public domains. Therefore, we recommend establishing an ELSI governance framework informed by TAPIC principles (transparency, accountability, participation, integrity, and capacity) (Greer et al., 2016). Figure 2 provides an overview of TAPIC and its application to biotechnology governance.

TAPIC identifies qualitative principles that inform responsive technology governance within this contested and uncertain environment. Recognizing that some regulatory challenges or limitations within the regulatory approval process will not be easy to overcome, the TAPIC framework seeks to promote greater coordination across biotechnology stakeholders, as well as foster a platform for flexible and adaptive action to prevent or mitigate risk early within the development process. Each of the five TAPIC principles should be addressed; they are designed to alleviate long-standing concerns within a government’s risk culture and facilitate a general environment that is amenable to safe, productive, and efficient research and innovation.

### 5.2 Safety-by-design to maximize technological progress while avoiding downstream pitfalls

To meet the core needs of safety, security, and ELSI for acceptable technology, early risk preparation and management can assure the public that companies understand the full scope of risk that their emerging technology might pose. Technology developers must invest in accurate threat and risk assessments and perform them early, often, and transparently. Doing so can reveal problems and conflicts early in project development and subsequently negate the need for future crisis interventions or reductions in capacity for biotechnology deployment.

Herein we propose a safety-by-design process for biotechnology development in which developers explicitly examines safety, security, and ELSI as part of the biotechnology innovation process. Specifically, safety-by-design is an operational goal where, through technical or procedural interventions, potential technology hazards are removed early in the development process and evaluated iteratively throughout development and commodification for ongoing risks to humans or the environment. For physical and biological sciences, safety-by-design may include, for example, developing “kill switches” that limit the potential for an engineered organism to proliferate over time and outside of a target deployment zone (thereby limiting unintentional exposure scenarios that could lead to permanent disruption of the natural local environment). Regulators can also contribute to safety-by-design by writing regulations that stipulate safeguards against undesirable endpoints (e.g., preventing irreversible or widespread introduction of engineered organisms into the environment). Such regulations would require direct input from experts in the biotechnology field. Additional regulations can mandate pre-market and post-market testing and evaluation requirements to identify unforeseen or unmitigated hazards throughout the research, development, and technology commodification process.

The intent of safety-by-design is to broaden, rather than restrict, the options available for biotechnology developers through demonstrable, technical, safety benchmarks, as well as expanding support from an accepting and informed consumer base and public. By allowing developers to frame and assess a technology’s implications early to identify and address risks of unacceptable outcomes or products, it is possible to streamline the innovation-to-commodification timeline, limit downstream hazards, alleviate expensive risk transfer requirements (e.g., various forms of insurance), and promote public confidence by demonstrating that biotechnology products are stringently tested and reviewed against conservative benchmarks before reaching the marketplace.

**FIGURE 3:**
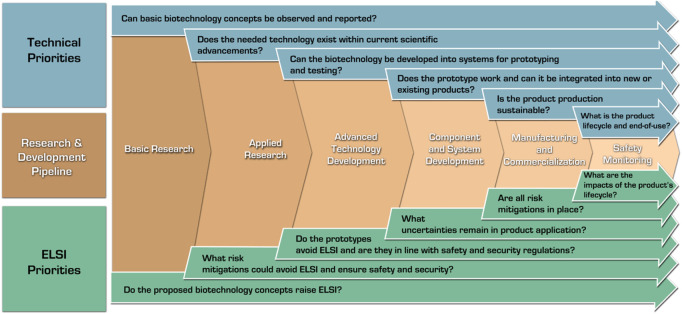
Safety-by-design in the research and development pipeline: ELSI and technical considerations.

Developers should apply safety-by-design early and often by engaging with the potential users of their products. Critically, safety-by-design is not a singular exercise, but requires developers, regulators, and policymakers to come together to understand *future* risk concerns as a biotechnology comes closer to the marketplace (e.g., could risk governance requirements become stricter in mitigating or preventing specific hazards, such as preventing unintended environmental release and proliferation of genetically modified material?). Figure 3 visualizes various guiding technical and ELSI exploration questions. As these questions address the core needs listed above and the general technological needs, the development process path narrows to the most beneficial applications.

## 6 Establishing a safety-by-design approach informed by TAPIC principles to incorporate ELSI in biotech modernization

Deploying living products on/in humans or in the environment carry the risks of negative ecological and human health impacts. Such products pose ethical concerns with environmental products potentially being viewed as “messing with nature,” while products for use on/in people may pose significant human subject research challenges. Not only is better risk assessment capability required for these two use cases, but communication regarding social implications, ethics, and governance safeguards must be transparently framed and articulated. Biotechnology deployment in any environment necessitates understanding biosafety and biosecurity risks, as well as the laws, international agreements and accords, and local cultural values and ethical considerations of the specific jurisdiction of deployment (see Figure 2 for more detailed information).

To foster safety-by-design for biotechnology modernization, there will be unique and escalating ELSI considerations based on the type of biotechnology and evolving realities of biotechnology acceptance and regulation. As biotechnology capabilities expand in their complexity (i.e., degree of control and efficiency of outcomes for an intended purpose) and health impact (i.e., potential to substantially and irreversibly impact human and environmental health), ELSI concerns may magnify due to perceptions of increased risk and/or ethical dilemmas. For example, ELSI concerns for biotechnology research with limited to no environmental deployment or human health application (e.g., biomanufacturing) will still be required to address multiple safety, security, and implications-based questions prior to commodification; these types of questions and requirements will only grow in number and in stringency in their evaluation for biotechnologies intended for widespread environmental deployment or human health applications (e.g., widely distributed biosensors, environmental gene drives, or engineered probiotics to improve gut health, among many others).

Many extant and growing ELSI challenges to emerging biotechnologies stem from long-standing debates from prior decades regarding research into genetically modified organisms (GMOs), such as with GMO agriculture (Cummings and Peters, 2022). Representative surveys note that the United States public does not generally feel informed about issues like synthetic biology, and they express moral reservations about the development and potential use of advanced biotechnologies (Akin et al., 2017). Much of the broader public perceives and interprets information about emerging biotechnologies through the lens of these prior debates, with additional concerns that are associated with their understanding of the advanced capabilities that can be leveraged from technologies, such as synthetic biology (Dragojlovic and Einsiedel, 2013; Aerni, 2021; Kuzma and Cummings, 2021). This includes public concern related to poorly characterized biotechnology risks (e.g., limited hazard characterization, exposure pathway benchmarking, or effects assessments) to human or environmental receptors. Likewise, significant discussion includes concerns pertaining to ethical (e.g., “who should bear the risk of testing these products?”), moral (e.g., “is biotechnology research violating the sanctity of life?”), and broader societal questions (e.g., “will biotechnology create a new privileged class of individuals with amplified physical/cognitive characteristics?”)—all points that were raised in previous generations of technological development. As such, government institutions and the public are already attuned to biotechnology discourse—often negatively and driven by older biological sciences (Trump et al., 2019; O’Mahony, 2020).

To improve biotechnology modernization prospects, it is essential to be mindful of continuing and entrenched ELSI debates, as well as the unique technical and social challenges stemming from ongoing and aspirational biotechnology research. Lessons from broader technology development indicate that the decisions to engage ELSI challenges today, alongside the trajectory of physical and life sciences research, will shape the nature and extent of ELSI challenges and roadblocks to modernization in the future (Mnyusiwalla et al., 2003; Fisher 2005; Renn and Roco 2006; Torgersen 2009; Roco et al., 2011; Greenbaum 2015; National Academies of Sciences, Engineering, and Medicine 2017; Bogner and Torgersen 2018; Trump et al., 2020a; Trump et al., 2020b). Institutionally, this includes the development of benchmarks and “gold standards” to characterize and evaluate hazard and exposure, as well as scalable regulatory guidance that “future-proofs” biotechnology governance in the light of unanticipated consequences of use (e.g., requiring developers to develop remediation agents or molecular “kill switches” for engineered organisms intended for environmental deployment).

Biotechnology has powerful capabilities to address societal needs, but miscalculations that produce damages could make other biotechnology deployments more difficult in the future. Addressing these considerations for biotechnology research and manufacturing requires diverse collaborators and cooperation across multiple stakeholders spanning academic, government, industry, and public domains. The TAPIC principles (transparency, accountability, participation, integrity, and capacity) (Greer et al., 2016) can inform best practices, build trust in organizations, and help maximize benefits of strategic interventions while minimizing undo risk. Adapting the TAPIC principles to the ELSI framework can systematically and quantifiably inform risk evaluations and enable standardization which ensures information needs are addressed while allowing for adaptability for individual technologies and organizational partnerships. Given the evolving nature of emerging biotechnologies, it is plausible that additional social, governmental, and scientific priorities will require additional requirements to be added to the TAPIC and ELSI principles.

Table 2 uses four brief case studies of biotechnology product classes to apply the TAPIC framework and highlight governance gaps. There are many similarities between the first and second case studies: biological systems producing specialty materials and chemicals, and data acquisition and analysis from biological systems. The latter differs in integrity, where more information is needed to characterize information hazards and biotechnology risks. The third and fourth case studies, respectively examine biologically living systems supporting non-living platforms and biologically living systems augmenting human performance. These present additional challenges; they both lack clear regulations and guidelines for pre-market and after-market reviews, pose a greater risk from information hazards, and latter has a lower level of collaboration between social scientists and governments, which imperil the technologies expansion if not adequately addressed. Table 2 provides additional information.

**TABLE 2:** Governance gaps across biotechnology product classes.

	Biological systems producing specialty materials and chemicals	Data acquisition and analysis from biological systems	Biologically living systems supporting non-living platforms	Biologically living systems augmenting human performance
	*Engineered microorganisms in biomanufacturing (for non-pathogenic materials)*	*Sensors with non-living* vs. *living components*	*Self-healing materials*	*Probiotics*
*Transparency*	• Existing laws, policies, regulations exist to serve as foundation for product specific policies but limited for future emerging technologies	• Existing laws, policies, regulations exist to serve as foundation for product specific policies but limited for future emerging technologies	• Products have high risk and uncertainty so require a broad risk communication and public education effort	• Products have very high risk and uncertainty so require a robust risk communication and public education effort
	• Regular reporting of biosafety risks or incidents, but require updates as technology advances	• Regular reporting of biosafety risks or incidents, but require updates as technology advances	• Extensive performance management, reporting, and assessment systems throughout entire product life, but require updates as technology advances	• Extensive performance management, reporting, and assessment systems for product and environment, but require updates as technology advances
	• Strong performance management, reporting, and assessment systems exist, but require updates as technology advances	• Strong performance management, reporting, and assessment systems exist, but require updates as technology advances	• Existing policy and legal framework can present roadblocks for future product specific laws and regulations	• Existing policy and legal framework can present roadblocks for future product specific laws and regulations
	• Require regulatory inspectorates and robust quality control systems which are currently lacking	• Require regulatory inspectorates and robust quality control systems which are currently lacking	• Require national and international watchdog committees	• Require national and international watchdog committees
	• Products have moderate risk and uncertainty and require usable public information about technology risks	• Products have moderate risk and uncertainty and require usable public information about technology risks	• Lack of clear regulations and guidelines for pre-market and after-market reviews	• Lack of clear regulations and guidelines for pre-market and after-market reviews
*Accountability*	• Existing conflict of interest policies and codes of conduct exist, but limited for future emerging, complex technologies	• Existing conflict of interest policies and codes of conduct exist, but limited for future emerging, complex technologies	• Lack of clear conflict of interest policies across agencies and organizations	• Lack of codes of conduct and clearly delineated conflict of interest policies at the regulatory level
	• Lack of protocols specifying ELSI objectives	• Lack of protocols specifying ELSI objectives	• Existing codes of conduct in medicine and genetics need to be adopted for future biotechnologies	• Unclear standards for safety across biotechnologies used with/in living systems
	• Established biosecurity and biosafety objectives and reporting mechanisms. But require updates as technology advances	• Established biosecurity and biosafety objectives and reporting mechanisms, but require updates as technology advances	• Existing medical and genetic standards for safety and ELSI objectives need to be adapted and built upon to develop for specific biotechnology applications	• Lack of clear standardized protocols (organizational, national, or international) specifying ELSI, biosecurity and biosafety objectives and reporting mechanisms
	• Limited after-market regulations	• Limited after-market regulations	• Established biosecurity and biosafety objectives and reporting mechanisms, but require updates as technology advances	• Require mechanisms to limit risk of information hazards
	• Existing dual-use reporting obligations for financing eligibility, but require updates as technology advances	• Existing dual-use reporting obligations for financing eligibility, but require updates as technology advances	• Require mechanisms to limit risk of information hazards	• Limited and fragmented pre-market and limited and fragmented or no after-market regulations
			• Limited and fragmented or no after-market regulations	• Existing dual-use reporting obligations for financing eligibility, but require updates as technology advances
			• Existing dual-use reporting obligations for financing eligibility, but require updates as technology advances	
*Participation*	• Some collaboration between social scientists (i.e., universities, academia) and government, but lack of specific laws, policies, regulations, and programs	• Some collaboration between social scientists (i.e., universities, academia) and government, but lack of specific laws, policies, regulations, and programs	• Some, but limited collaboration between social scientists (i.e., universities, academia) and government	• Limited to no collaboration between social scientists (i.e., universities, academia) and government
	• Limited to no public participation in policy or regulation creation	• Limited to no public participation in policy or regulation creation	• Limited to no public participation in policy or regulation creation	• Limited to no public participation in policy or regulation creation
	• Some, but limited official governmental advisory committees established or some clear guidelines exist for which agency has regulatory authority	• Some, but limited official governmental advisory committees established or some clear guidelines exist for which agency has regulatory authority	• Limited or no established governmental advisory committees for biotechnologies in product class (i.e., synbio)	• Limited or no established governmental advisory committees for biotechnologies in product class (i.e., synbio)
	• Growing mechanisms to increase clarity in how decisions are made across government agencies and between non-government stakeholders, but more transparency and citizen-involvement is needed	• Some clarity in how decisions are made across government agencies and between non-government stakeholders, but more transparency and citizen-involvement is needed	• Limited clarity in how decisions are made across government agencies and between non-government stakeholders	• Lack of clarity in how decisions are made across government agencies and between non-government stakeholders
*Integrity*	• Clear personnel policies in private companies and in research facilities, but require updates as technology advances	• Clear personnel policies in private companies and in research facilities, but require updates as technology advances	• Clear personnel policies in private companies and in research facilities, but require updates as technology advances	• Clear personnel policies in private companies and in research facilities, but require updates as technology advances
	• Some, but limited legislative mandates or budgets for synthetic biotechnology development, limited to existing biotechnologies	• Some, but limited mandates or budgets for synthetic biotechnology development, limited to existing biotechnologies	• Some, but limited legislative mandates or budgets for biotechnology development, limited to medical applications	• No clear legislative mandates or budgets for synthetic biotechnology development
	• Need for standardization on governance procedures	• Need for standardization on governance procedures	• Need for standardization on governance procedures	• Need for standardization on governance procedures
	• Some, but limited internal and external risk audits occurring	• Some, but limited internal and external risk audits occurring	• Need for internal and external risk audits	• Need for internal and external risk audits
	• Limited public sector career trajectories with most solid, well-rewarded career trajectories in private sector	• Limited public sector career trajectories with most solid, well-rewarded career trajectories in private sector	• Lack of public sector career trajectories for biotechnology development (rewarding service over profit)	• Lack of public sector career trajectories for biotechnology development (rewarding service over profit)
	• Resources that educate researchers, students, and employees about information hazards and biotechnology risks exist, but require updates as technology advances	• More resources needed to educate researchers, students, and employees about information hazards and biotechnology risks	• More resources needed to educate researchers, students, and employees about information hazards and biotechnology risks	• More resources needed to educate researchers, students, and employees about information hazards and biotechnology risks
*Capacity*	• Strong research and analysis capacity, but future investments into research need to be maintained	• Strong research and analysis capacity, but future investments into research need to be maintained	• Growing academic collaborations with government in biotechnology, but limited for synbio, translational science, and social science	• Growing academic collaborations with government in biotechnology, but limited for synbio and social science
	• Existing and growing technical policy capacity, but require updates as technology advances	• Existing and growing technical policy capacity, but require updates as technology advances	• Limited but growing technical policy capacity, but need for increased educational opportunities for social science of biotech	• Limited but growing technical policy capacity, but need for increased educational opportunities for social science of biotech
	• Limited investment into complementary fields that could increase capacity	• Limited investment into complementary fields that could increase capacity	• Limited investment into complementary fields that could increase capacity	• Limited investment into complementary fields that could increase capacity
	• Limited manufacturing capabilities due to economies of scale and expensive barriers to entry for small businesses	• Limited manufacturing capabilities due to economies of scale and expensive barriers to entry for small businesses	• Limited manufacturing capabilities due to economies of scale and expensive barriers to entry for small businesses	• Limited manufacturing capabilities due to economies of scale and expensive barriers to entry for small businesses
	• Challenging international and national regulatory and legal framework which could hinder biotechnology development	• Challenging international and national regulatory and legal framework which could hinder biotechnology development	• Challenging international and national regulatory and legal framework which could hinder biotechnology development	• Limited to no national laboratory or research facilities dedicated to synthetic biology
				• Challenging international and national regulatory and legal framework which could hinder biotechnology development

By implementing these principles as foundational guidance for achieving safety by design in biotechnology, governments can improve technical and ELSI exploration while averting deleterious events that would culminate in negative outcomes, optics, and public perceptions. As biotechnology inquiry progresses, renewed considerations of infrastructure and facility needs must also be raised.

## 7 Discussion

Safety-by-design can assess information security through all stages of development to prevent information hazards (e.g., access of critical information among nefarious actors) and ensure that data management plans prevent disclosure of private data. This includes data particular to individuals (e.g., genomic heritage) as well as biotechnology developers and manufacturers, for whom, information disclosure would cease competitive advantage to bring applications to market. Information security must include individual and population-level safeguards, such as considerations of individual privacy as well as limitations on potential information hazard transfer.

To succeed upon implementation, however, safety-by-design requires improvements throughout the technology governance process. Where many emerging biotechnology applications are characterized by uncertain and potentially grave hazards, fostering risk-based and ELSI-centered risk assessment, management, and communication structures will be a departure from the conventional governance of longstanding environmental and consumer product practices. This framework will need to resolve several political and institutional challenges. For instance, it is unclear which regulatory and funding agencies have a statutory responsibility to craft and implement ELSI standards, as well as how to benchmark success or failure for the standards. It is hoped that addressing social values early in the development process, and incentivizing governance institutions to foster a transparent safety culture, will forestall further reluctance to embrace biotechnology products by minimizing health and safety risk.

In addition, there are existing biosafety and biosecurity frameworks, as well as laws and regulations that apply to hazardous materials that can be leveraged to capture safety-by-design operating practices. Emerging capabilities (e.g., data acquisition and analysis from biological systems) possess a larger scope of concerns, including the need to improve upon significant gaps in risk assessment capabilities. However, ELSI objectives are not always consistent with each other, requiring tradeoffs among ELSI issues themselves that may be values-based. For example, there are challenges in balancing data access and analysis with biosecurity and privacy protection.

While ELSI is a long-standing issue in academia, translating it into policy is a more recent endeavor. The frameworks reported here can be a platform to stimulate future scientific enquiry and empirical validation. For instance, a future study might evaluate how actors might seek to abuse the system by prioritizing their own impacts or risks, real or imagined, over the general benefits to broader society. Other issues such as misinformation and power imbalances in public discourse that amplify the voices advocating against innovation can bias discussions about social benefits in public discourse and policy.

Going forward, the ELSI framework must include the presence of bad faith actors seeking to game the ELSI framework to advance their own particular interests without having any interest in coming to an optimal societal outcome. Thus, technology developers have two choices: engage with those actors in the ELSI framework, potentially under increasingly stringent limitations, or engage with policymakers with the jurisdiction, willingness, and political capital to support technological applications based on those policymakers’ own ELSI criteria, notwithstanding the objections of bad faith or overly risk-averse actors. However, the designation of such actors is delicate: stakeholder objections may reflect historical injustices that have raised the specter of authoritarian overreach that will harm the broader population (Gibson, 2005). Careful balance is needed to respect the views of risk-averse populations without letting them unduly derail projects that the broader population finds acceptable and beneficial. The ELSI framework will assist in identifying major deviations in viewpoints and helping decision makers understand the costs and benefits that are most salient to their goals. Indeed, the ELSI framework will be necessary to ensure that all concerns have been considered, explored, and satisfied to the threshold set by policymakers.

True safety-by-design can only be achieved through such robust considerations requiring well thought-out and executed strategies to mitigate risks across all stages of development, use, and end of life. Biotechnology governance must be thoughtfully crafted, implemented, and adapted early in the technology development process if modernization plans, timelines, and economies are to be satisfactorily achieved. Accomplishing this requires balancing risks and benefits, including for hazards with poorly characterized properties and uncertain hazards. Mechanisms like TAPIC can help balance the life sciences and social sciences of biotechnology, although every country and discipline will interpret and manage risk according to local customs, politics, and institutional incentives.

There are tremendous political, technological, and commercial opportunities at stake for those competing in the biotechnology development space. As such, and despite the risk and ELSI challenges facing the field, national and industry practitioners will pursue biotechnology to achieve strategic objectives. Without a cohesive governance structure, differing incentives will drive uneven technology development and exacerbate social concerns pertaining to ethics, values, and equitable distribution of benefits and risks. Where the field will continue to expand in complexity and product prominence, we anticipate the need for safety-by-design in technology readiness level benchmarking will become increasingly required to maintain a robust bioeconomy, as well as prevent diverging risk culture from making countries and industries incompatible with their societies or one another (Thormann et al., 2021; Pascoli et al., 2022).

## Data Availability

The original contributions presented in the study are included in the article/Supplementary Material, further inquiries can be directed to the corresponding author.
